# Human Parvovirus 4 in Kidney Transplant Patients, France

**DOI:** 10.3201/eid1411.080862

**Published:** 2008-11

**Authors:** Philippe Biagini, Bertrand Dussol, Mhammed Touinssi, Philippe Brunet, Christophe Picard, Valérie Moal, Mourad Belhouchet, Pierre Gallian, Jean-François Cantaloube, Houssam Attoui, Yvon Berland, Philippe de Micco

**Affiliations:** Etablissement Français du Sang Alpes-Méditerranée, Marseille, France (P. Biagini, M. Touinssi, C. Picard, P. Gallian, J.-F. Cantaloube, P. de Micco); Université de la Méditerranée, Marseille (P. Biagini, M. Touinssi, P. Gallian, J.-F. Cantaloube, P. de Micco); Centre Hospitalier Universitaire, Marseille (B. Dussol); Hôpital de la Conception, Assistance Publique de Marseille, Marseille (P. Brunet; V. Moal, Y. Berland); Institute for Animal Health, Pirbright, UK (M. Belhouchet, H. Attoui)

**Keywords:** PARV4, blood, transplant, kidney, hemodialysis, blood donors, parvovirus, letter

**To the Editor:** Human parvovirus 4 (PARV4) is a recently identified virus, distantly related to already known members of the family *Parvoviridae* that affect humans and animals. Initially, PARV4 was characterized in the blood of a North American patient who had acute viral syndrome; a sequence-independent amplification approach was used (*1*). Molecular analysis of the viral prototype genome (5,268 nt) identified 2 large, nonoverlapping open reading frames (ORFs) and showed limited homology with human parvovirus B19 (<30% aa similarity). Subsequent phylogenetic analyses have shown that at least 2 genotypes are identifiable, differing by ≈8% at the nucleotide level (*2*).

The first prevalence studies, performed mainly in North America and the United Kingdom, reported finding the virus in plasma samples from febrile patients who had symptoms resembling those of acute HIV infection (6%), from cadavers of hepatitis C RNA–positive intravenous drug users (30%), and in plasma donations from healthy blood donors (5% pooled, 2% individual) (*3*–*5*). PARV4 was also identified in clotting factor VIII concentrate and in plasma pools negative for parvovirus B19 DNA (*5*,*6*). This new virus appears to not be restricted solely to blood samples; it has been already identified in bone marrow, in various autopsy tissue samples from patients with AIDS, and in liver tissues of persons with liver dysfunctions (*7*–*9*). Typical amounts of PARV4 DNA identified in the various samples tested ranged from <500 to >10^6^ copies/mL.

We investigated PARV4 DNA in plasma samples collected from cohorts of 378 inpatients and 192 healthy blood donors from southeastern France during 2007: 164 kidney transplant patients (55 women, 109 men; mean age 51 ±14 years; mean duration of transplantation 37 ±30 months), 214 hemodialysis patients (88 women, 126 men; mean age 65 ±15 years; mean duration of dialysis 30 ±28 months), and 192 voluntary blood donors (86 women, 106 men; mean age 40 ±21 years).

Blood samples were collected in vacuum tubes (Vacutainer SST; Becton Dickinson, Meylan, France) and then centrifuged. Plasma aliquots were stored at –80°C before DNA extraction. Nucleic acids were extracted from 1-mL volumes of plasma by using a nucleic acid extraction machine (MagNA Pure LC, Roche Diagnostics, Meylan, France) and eluted into 50-μL volumes. Samples were screened for PARV4 DNA by real-time PCR (StepOne Plus, Applied Biosystems, Courtaboeuf, France) by using a consensus TaqMan PCR system composed of conserved primers and a fluorogenic hydrolysis probe located on the ORF2 of the viral genome (*5*). Amplification reactions were performed by using 5 μL of extracted nucleic acids with the TaqMan Fast Universal PCR kit (Applied Biosystems) in a final volume of 20 μL. The amplification conditions were 95°C for 20 s, followed by 50 cycles of 95°C for 1 s and 60°C for 20 s. Using dilutions of a synthetic template corresponding to the target sequence (103 nt), we estimated the sensitivity of the TaqMan assay to be 10 copies of PARV4 DNA.

Positive results were obtained from 5 blood samples, all from kidney transplant patients (5/164; 3.05%). Real-time PCR products were cloned, sequenced, and compared with sequences of PARV4 already deposited in databases. They exhibited 100% nucleotide identity in this ORF2 region with the PARV4 prototype isolate (GenBank accession no. AY622943). The titer of PARV4 DNA in the positive samples was low and did not exceed 500 copies/mL plasma. The 5 patients (1 woman and 4 men, mean age 48 ±18 years, mean duration of transplantation 25 ±21 months, no heterologous blood transfusions) did not show evidence of specific biological or clinical dysfunctions.

Whether these 5 patients were infected by kidney graft was impossible to determine because kidney transplant tissue samples were unavailable for analysis. However, 2 blood samples were available for retrospective analysis for 1 patient (male, 66 years), who was PARV4 positive at 4 months after transplant. These samples, collected 1 and 2 months before transplant, were negative for PARV4 DNA, which suggests possible transmission of the virus by the transplanted organ or reactivation of a latent infection resulting from immunosuppressive treatments.

PARV4 DNA was not detected in any persons in the 2 other cohorts: hemodialysis patients and voluntary blood donors. Investigations of larger cohorts and/or analyses of plasma pools, using optimized molecular approaches, are required for a better understanding of the diffusion of PARV4 in France.

In summary, we found PARV4 in the blood of transplant patients and determined that for 1 of these patients, PAR4 was present only after the transplant procedure. The natural history and clinical features of this new parvovirus remain largely unknown. Further investigations to elucidate the mode of transmission and the potential effect of PARV4 infection in this category of patients are urgently needed.
